# Preclinical evaluation of lime juice as a topical microbicide candidate

**DOI:** 10.1186/1742-4690-5-3

**Published:** 2008-01-11

**Authors:** Patricia S Fletcher, Sarah J Harman, Adrienne R Boothe, Gustavo F Doncel, Robin J Shattock

**Affiliations:** 1 St George's University of London, UK; 2 CONRAD, Eastern Virginia Medical School, USA

## Abstract

**Background:**

The continued growth of the global HIV epidemic highlights the urgent need to develop novel prevention strategies to reduce HIV transmission. The development of topical microbicides is likely to take a number of years before such a product would be widely available. This has resulted in a call for the rapid introduction of simpler vaginal intervention strategies in the interim period. One suggested practice would be vaginal douching with natural products including lime or lemon juice. Here we present a comprehensive preclinical evaluation of lime juice (LiJ) as a potential intervention strategy against HIV.

**Results:**

Pre-treatment of HIV with LiJ demonstrated direct virucidal activity, with 10% juice inactivating the virus within 5 minutes. However, this activity was significantly reduced in the presence of seminal plasma, where inactivation required maintaining a 1:1 mixture of neat LiJ and seminal plasma for more than 5 minutes. Additionally, LiJ demonstrated both time and dose-dependent toxicity towards cervicovaginal epithelium, where exposure to 50% juice caused 75–90% toxicity within 5 minutes increasing to 95% by 30 minutes. Cervicovaginal epithelial cell monolayers were more susceptible to the effects of LiJ with 8.8% juice causing 50% toxicity after 5 minutes. Reconstructed stratified cervicovaginal epithelium appeared more resilient to LiJ toxicity with 30 minutes exposure to 50% LiJ having little effect on viability. However viability was reduced by 75% and 90% following 60 and 120 minutes exposure. Furthermore, repeat application (several times daily) of 25% LiJ caused 80–90% reduction in viability.

**Conclusion:**

These data demonstrate that the virucidal activity of LiJ is severely compromised in the presence of seminal plasma. Potentially, to be effective against HIV *in vivo*, women would need to apply a volume of neat LiJ equal to that of an ejaculate, and maintain this ratio vaginally for 5–30 minutes after ejaculation. Data presented here suggest that this would have significant adverse effects on the genital mucosa. These data raise serious questions about the plausibility and safety of such a prevention approach.

## Background

Women are increasingly bearing the brunt of the global HIV epidemic, accounting for 50% of cases worldwide and >67% of cases in sub-Saharan Africa where three times more 15–24 year old women are infected than men [1]. The mantra of "abstinence, faithfulness and condoms" appears to be failing these vulnerable groups where men often refuse to use condoms and faithfulness only works if practiced by both partners [2]. The lack of alternative protection options available to women has led to the use of traditional practices such as vaginal douching with water, soap or acidic solutions in the belief that this may prevent HIV infection.

For an intervention strategy against HIV transmission to be effective it needs to fulfil criteria associated with cost, availability, acceptability, safety and efficacy [3,4]. The urgent need for the development of female-initiated strategies to prevent HIV-1 transmission has been the basis for international efforts to develop vaginal microbicides [4]. However, the timelines for the development of an effective microbicide (5–10 years) have led some to question whether simpler strategies using readily available natural products such as limes or lemons, might allow a more rapid introduction of a vaginal intervention strategy that could prevent infection even if only partially effective. Limes are cheap and readily accessible throughout all tropical and temperate regions of the globe [5], and thus are probably accessible to the majority of the world's population. Therefore they most likely fit the first three criteria of an effective intervention strategy (cheap, available, acceptable), however little is known about the other criteria – safety and efficacy.

The hypothesis that lime/lemon douching might prevent HIV transmission is based upon existing data showing that a pH <4.5 is sufficient to inactivate HIV *in vitro *[6]. Therefore, maintenance of a low pH (<4.0) has been the basis of several intervention strategies, specifically the development of acid buffering gels including BufferGel [7,8], which is currently in phase IIb clinical trials, and ACIDFORM [9], currently in phase I clinical trials. Recent data, however, indicate that non-clade B primary HIV-1 isolates may be less susceptible to low pH than the lab-adapted clade B viruses used in previous studies [10].

There is a long reported history of African women douching with lime juice (LiJ), lemon juice (LeJ), vinegar or acidic soft drinks in the belief that it may prevent pregnancy and/or sexually transmitted diseases (STDS) [5]. This suggests that should such practices be effective, they could be rapidly implemented. However the frequency and geographical distribution of such practices across Africa and other areas of the world with high HIV prevalence has not been systematically evaluated. More importantly, the impact of such practices on HIV transmission rates (positive or negative) has not been assessed. A recent survey of female sex workers (FSW) in the city of Jos, Nigeria reported that up to 80% of them regularly used LiJ/LeJ douches either before or after sex, and of those 68.6% used lime, 19.6% lemon and 11.8% used both [11]. A previous pilot study on a similar population of commercial FSW has shown that the most common method of using LiJ is to mix the juice from 1–4 limes with 1–4 teaspoons of water and douche with the resulting solution, but practices range from mixing the juice of one lime with one cup of water to mixing the juice of four limes with one teaspoon of water [12].

As LiJ has been evaluated in phase I clinical trials to assess its safety as a potential intervention strategy against HIV transmission, and its use may have already been adopted by women at risk of HIV infection, we have undertaken preclinical *in vitro *studies to assess the potential safety and efficacy of LiJ using cellular and *ex vivo *mucosal tissue models.

## Results

### Virucidal activity of lime juice

**Figure 1:**
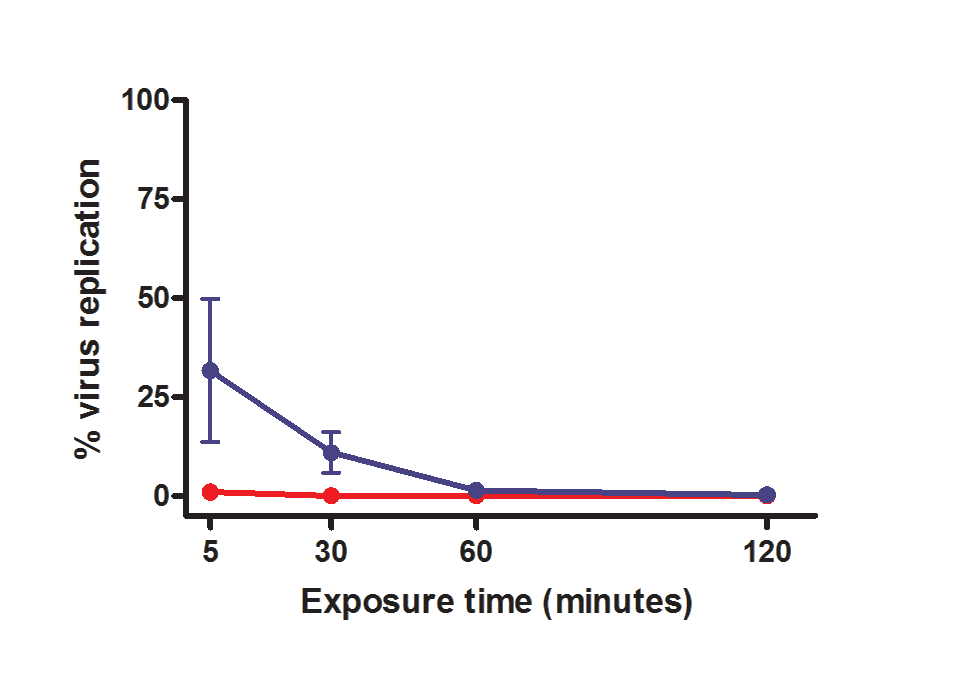
**Virucidal activity of lime juice**. HIV-1_BaL _(10^5 ^TCID_50_) was exposed to 5% (blue circles) or 10% (red circles) LiJ (diluted using RPMI 10% FBS) for 5 – 120 minutes. This HIV/juice mix was then diluted 1/10 with RPMI 10% and applied to human cervicovaginal explants. Following 2 hours, explants were washed to remove juice and excess virus, and cultured for 10 days with 50% media feeds every 2–3 days. HIV-1 infection was determined by measurement of p24 Ag release into culture supernatants. Data shown are expressed as percentage infection (when compared to an untreated virus control) and represent the mean ± SEM of n = 3 independent tissue donors where each condition was tested in triplicate.

To determine whether LiJ exhibited virucidal properties, HIV-1_BaL _was pre-treated with 5 or 10% LiJ (diluted using RPMI 1640 + 10% fetal bovine serum [RPMI 10%]) for 5, 30, 60 or 120 minutes prior to application onto human cervicovaginal tissue explants. To minimise the toxicity of LiJ to the cultured tissue, the virus/LiJ mixture was diluted 1/10 using RPMI 10% prior to tissue exposure. Following a 2 hour exposure to virus/LiJ, cervicovaginal tissue was washed and cultured for 10 days when viral infection was determined by the release of p24 antigen in the culture supernatants. To ensure that any lack of infection in cervicovaginal tissue was not due to LiJ toxicity, additional explants were exposed to equivalent LiJ concentrations for 2 hours and assessed for viability in parallel. Those concentrations of LiJ demonstrating more than 25% toxicity were not evaluated for HIV infection. On this basis, concentrations above 10% (i.e., 1% following the 1/10 dilution performed prior to exposure) could not be evaluated due to their significant toxic effects on cervicovaginal tissue (data not shown). At 5%, LiJ was seen to exhibit virucidal activity in a time-dependent manner, needing 60 minutes to completely inhibit viral infectivity (Figure 1). Five minutes, however, was sufficient for 10% LiJ diluted in culture medium to completely inactivate the virus.

### Virucidal activity in the presence of semen

**Figure 2:**
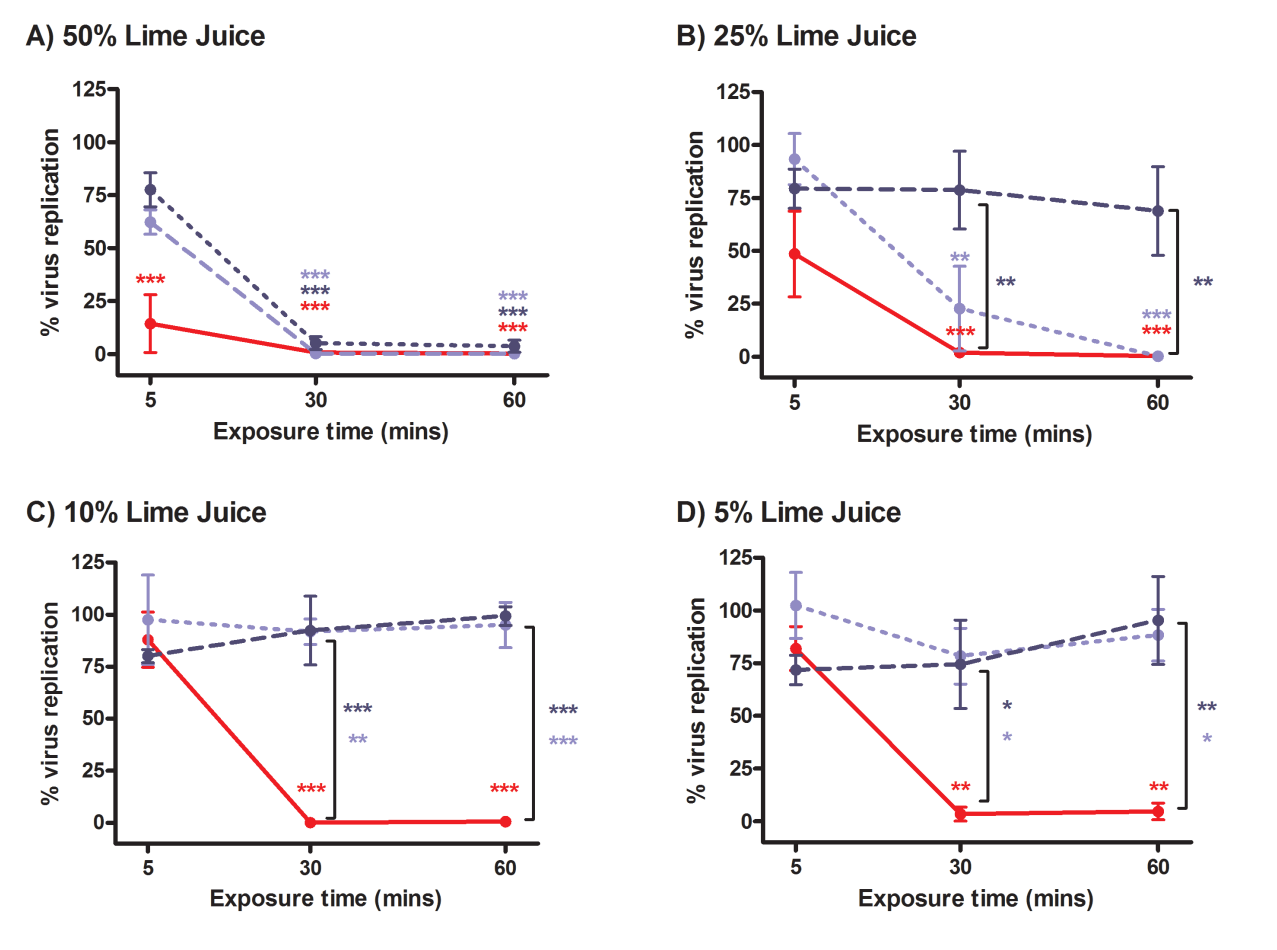
**Virucidal activity of lime in the presence of semen**. Immobilised HIV-1_RF _(using poly L-lysine capture) was treated with LiJ (diluted in normal saline) in the absence (red circles/solid line) or presence of seminal plasma (50% seminal plasma: dark blue circles/dashed line; 25% seminal plasma: light blue circles/dotted line) for 5, 30 or 60 minutes. Juice and semen were then removed by washing and immobilised virus cultured with C8166 T cells for 7 days. Viral replication was determined by measurement of RT in culture supernatants. LiJ concentrations tested were: A) 50%; B) 25%; C) 10%; and D) 5%. Data shown are expressed as percentage infection (when compared to an untreated virus control) and represent the mean ± SEM of n = 3 (25% seminal plasma) or n = 5 (50% or no seminal plasma) independent experiments where each condition was tested in triplicate. Statistical analysis (ANOVA with Bonferroni post tests) was performed comparing LiJ treated samples and an untreated viral control, and comparing samples in the presence (2 concentrations) or absence (LiJ only) of seminal plasma. Those conditions causing statistically significant differences are marked with asterisks (* p < 0.05; ** p < 0.01; *** p < 0.001) and are colour coded (red: juice only; dark blue: LiJ with 50% seminal plasma; light blue: LiJ with 25% seminal plasma).

Although LiJ was shown to have virucidal activity against HIV-1 infection of cervicovaginal explants, it was important to determine the virucidal activity in the presence of semen, the natural carrier of the virus during sexual transmission. This was completed using T cells and microplate-immobilised virus. Immobilised HIV-1_RF _was treated with LiJ in the absence or presence of 50 or 25% seminal plasma (SP) for 5, 30 or 60 minutes. Following LiJ/SP removal by washing, virus was then cultured with C8166 T cells for 7 days when viral replication was determined by viral reverse transcriptase (RT) activity in culture supernatants. As had been observed using cervicovaginal explants, LiJ diluted in saline (0.9% NaCl) was virucidal in both a time and dose-dependent manner, with ≥ 5% LiJ inactivating all virus after 30 minutes of incubation (Figure 2). However, in the presence of SP, virucidal activity was significantly reduced. In the absence of SP, 50% LiJ inactivated 80% of the virus within 5 minutes. However, in the presence of 25 or 50% SP, virus inactivation required LiJ exposure for 30 minutes to have the same effect. This effect was more noticeable with lower concentrations of LiJ, with the virucidal activity of ≤ 25% LiJ being eliminated by 50% SP. Furthermore, the presence of 25% SP significantly prevented any virucidal activity of ≤ 10% LiJ. Only a 30–60 minute treatment of virus with 50% LiJ in the presence of 25 or 50% SP was sufficient to completely inhibit HIV-1_RF _infection of T cells.

### Toxicity of lime juice on human genital tissue

**Figure 3:**
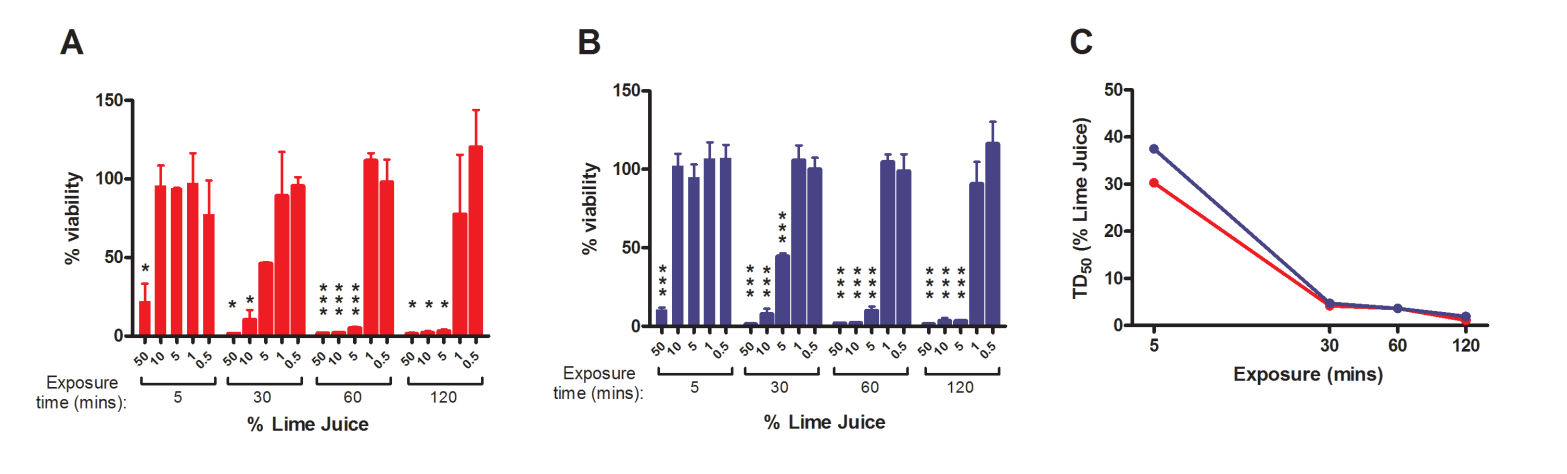
**Toxicity of lime juice on cervicovaginal and penile tissue**. (A) Human cervicovaginal and (B) penile tissue explants were exposed to LiJ (diluted using normal saline) for 5, 30, 60 or 120 minutes. Juice was then removed by washing and tissue viability assessed by the method of MTT dye reduction. Data shown are expressed as percentage viability (when compared to an untreated control) andrepresent the mean ± SEM of n = 2 (cervicovaginal) or n = 3 (penile) independent tissue donors where each condition was tested in triplicate. Statistical analysis (ANOVA with Bonferroni post tests) was performed comparing LiJ treated samples with an untreated control. Those conditions causing statistically significant differences are marked with an asterisk (* p < 0.05; ** p < 0.01; *** p < 0.001). (C) The 50% toxic dose (TD_50_) of LiJ was determined using non-linear regression analysis for cervicovaginal (red circles) and penile (blue circles) tissue.

As the initial determinations of virucidal activity using genital tissue had demonstrated that LiJ exhibited a potential toxic effect, any detrimental effects attributable to LiJ application were evaluated further. Cervicovaginal and penile tissue explants were exposed to LiJ (0.5–50% diluted in normal saline) for 5, 30, 60 or 120 minutes and toxicity determined using the principle of MTT dye reduction. Exposure to LiJ exhibited significant dose and time-dependent toxic effects on genital tissue with similar effects observed with both cervicovaginal (Figure 3A) and penile tissue explants (Figure 3B). In general, increased exposure times resulted in decreased 50% toxic dose (TD_50_) values (Figure 3C), whilst low concentrations (0.5–1%) of LiJ appeared non-toxic to genital tissue, even following 120 minute exposure. However, as little as 5% LiJ caused significant toxicity to genital tissue following exposure times of 30 minutes or more. Furthermore, a 5 minute exposure to 50% LiJ caused a 75–90% reduction in viability, and >95% reduction following a 30 minute exposure period in both cervicovaginal and penile tissue explants.

### Toxicity of lime juice to cervical epithelial cells

**Figure 4:**
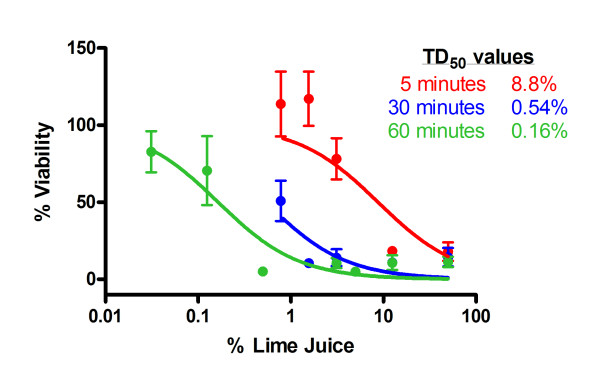
**Toxicity of lime on cervical epithelial cells**. Cervical epithelial cells (ME180) were exposed to LiJ (diluted using normal saline) for 5 (red circles), 30 (blue circles) or 60 (green circles) minutes. Juice was then removed by washing and tissue viability assessed by the method of MTT dye reduction. Data shown are expressed as percentage viability (when compared to an untreated control) andrepresent the mean ± SEM of n = 4 independent experiments where each condition was tested in quadruplicate. The 50% toxic dose (TD_50_) of LiJ was determined using non-linear regression analysis.

The potential toxicity caused to the epithelium of the cervix was evaluated using the cervical epithelial cell line ME180. As previously, cells were exposed to LiJ diluted in normal saline for 5, 30 or 60 minutes when cell viability was then determined. Exposure of cervical epithelial cells to LiJ again caused a significant toxic effect that was both time and dose-dependent (Figure 4). Whilst treatment for only 5 minutes caused a 50% reduction in viability at 8.8% juice, this decreased to 0.54% following 30 minutes, and 0.16% following a 60 minute exposure.

### Toxicity of lime juice application on the stratified cervicovaginal epithelium

**Figure 5:**
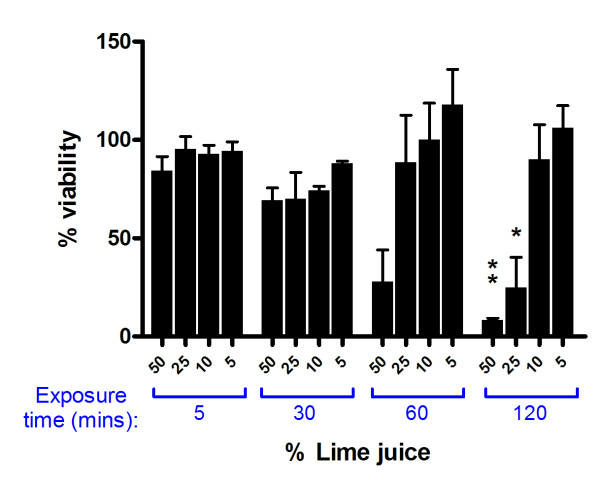
**Toxicity of lime juice following topical application onto reconstructed cervicovaginal epithelia**. Reconstructed cervicovaginal epithelial tissues (MatTek Corporation) were exposed to LiJ (diluted using normal saline) for 5, 30, 60 or 120 minutes. Juice was then removed by washing and tissue viability assessed by the method of MTT dye reduction. Data shown are expressed as percentage viability (when compared to an untreated control) andrepresent the mean ± SEM of n = 3 independent experiments. Statistical analysis was completed using ANOVA with Bonferroni post tests and statistically significant changes marked with * (p < 0.05) or ** (p < 0.01).

To evaluate the effect of topical application of LiJ onto an intact, stratified cervicovaginal epithelium, investigations were completed using reconstructed cervicovaginal epithelial cultures (MatTek Corp). Although the stratified cervicovaginal epithelium appeared to be less susceptible than mucosal genital tissue to the toxic effects of LiJ (diluted in saline) following topical application, dose and time-dependent effects were also observed (Figure 5). Whilst there were no obvious signs of toxicity following a 5 or 30 minute treatment period with up to 50% LiJ, a significant reduction in viability was observed following 60 minutes, with 50% LiJ causing a 75% reduction in viability. Furthermore, a 2 hour exposure to 25% LiJ resulted in 65% reduction in viability and 50% LiJ caused 90% toxicity. In contrast to genital tissue explants, a single topical application of 5 or 10% LiJ to the stratified cervicovaginal epithelium did not cause any obvious toxicity even when exposure occurred for 2 hours.

**Figure 6:**
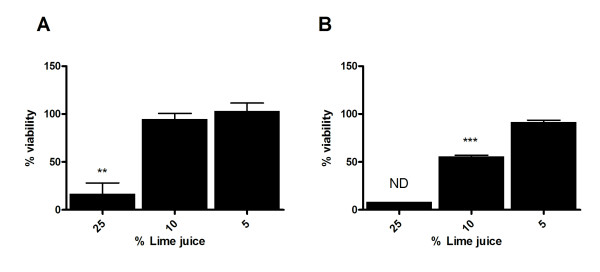
**Toxicity of lime juice following repeat topical application onto a reconstructed cervicovaginal epithelium**. Reconstructed cervicovaginal epithelial tissues (MatTek Corporation) were repeatedly exposed to LiJ (diluted using normal saline) for 30 minutes. Exposure occurred either: (A) once daily for 5 days; or (B) 5 times in one day (30 minute exposure, wash, culture for 1 hour then repeat exposure). After the final exposure, juice was removed by washing and epithelial viability assessed by the method of MTT dye reduction. Data shown are expressed as percentage viability (when compared to an untreated control) andrepresent the mean ± SEM of n = 2 independent experiments where each condition was tested in triplicate, except for 25% LiJ exposed repeatedly in one day which was only tested in one experiment. Statistical analysis was performed (ANOVA with Bonferroni post tests) to compare LiJ treated wells and an untreated control, and statistically significant changes are marked with ** (p < 0.01); or *** (p < 0.001). ND: not determined (due to only one dataset).

To investigate whether an intact stratified cervicovaginal epithelium would be more susceptible to the toxic effects of LiJ following multiple applications, reconstructed cervicovaginal epithelial cultures were repeatedly exposed to a topical application of LiJ. Multiple exposures occurred either over one day (five 30 minute treatments followed each time by a 60 minute culture period in the absence of LiJ), or once a day for 5 days (30 minute exposure each day, followed by overnight culture in the absence of LiJ). Following either repeat treatment regime, the stratified cervicovaginal epithelium appeared to be more susceptible to the toxic effects of LiJ in a dose and time-dependent manner (Figure 6). Significant toxicity was observed following repeat application, with 25% LiJ causing 80–90% reduction in viability. Whilst repeated application of 10% LiJ throughout the course of one day caused a 60% reduction in epithelial viability, the same LiJ concentration appeared non-toxic when applied once daily for 5 consecutive days. In neither treatment regime was 5% LiJ seen to cause any detrimental effects.

## Discussion

It has long been recognized that HIV is inactivated by low pH (<4.0) [6,13-15]. Therefore the reduction of vaginal pH by LiJ provides the basis for its potential use as an anti-HIV intervention strategy. However SP is known to have significant buffering capacity to overcome the low vaginal pH that would be hostile to sperm viability [16]. Such neutralization of vaginal pH has been postulated as a possible co-factor for sexual transmission of HIV [17]. Furthermore, it should be noted that an evaluation of 19 varieties of lime found worldwide (obtained from the USDA Repository in California), demonstrated significant variation in colour, size, and the volume and pH of extracted juice (data not shown). In particular, "sour" limes had a very different pH to "sweet" limes (pH 2.7 ± 0.2 versus 6.2 ± 0.2; data not shown).

A previous report has demonstrated that 20% LeJ was sufficient to reduce the pH of SP from 8.4 to 4.1 [18], supporting its potential use as an acidic douche to reduce pregnancy and STD transmission. Indeed, we also observed that 50% sour LiJ significantly reduced the pH of semen from pH 7.6 to 3.2 (data not shown). Furthermore, 20% LeJ or LiJ was sufficient to immobilise sperm within 1 minute [18] (Doncel, unpublished), suggesting that both LeJ and LiJ could have spermicidal activity *in vivo*. LiJ has previously been reported to exhibit potent virucidal activity against HIV, with 20% LiJ inactivating 80% of HIV within only 2 minutes and 10% juice in 60 minutes [19]. Data presented here support these findings, with concentrations of 50% and 5% LiJ able to inactivate HIV *in vitro *within 5 and 30 minutes, respectively. However, whilst others have suggested that LiJ retains its activity in the presence of SP [19], we found its antiviral activity to be severely reduced by this fluid (25 or 50% SP), requiring the maintenance of a 1:1 mixture of neat LiJ and neat SP for more than 5 minutes (and up to 30 minutes) to fully inactivate HIV. The reduction in the inhibitory activity of LiJ in the presence of SP is mostly linked to its buffering capacity, suggesting that the main antiviral activity of LiJ is mediated by low pH. This is in agreement with previous studies that have shown LiJ, unlike pomegranate juice, has no effect on gp120-CD4 interaction [20].

Whilst the use of LiJ may appear to be acceptable to Nigerian FSW [11], data from clinical studies have already raised questions as to its safe use in areas of high HIV prevalence. Nineteen percent of women in the Nigerian cohort reported pain following the use of LiJ/LeJ [11], however no information was acquired about less "serious" but uncomfortable side effects such as vaginal dryness, itching or burning. Application of LiJ via tampon insertion or douching has been evaluated under more controlled conditions and compared with application of water alone. Both LiJ and water were found to cause mild and transient side effects in 70% of women, including vaginal dryness, itching and burning, but burning and dryness occurred more frequently in women using 20% LiJ [21]. Evaluation of higher LiJ concentrations (50 and 100% via douching or tampon application) found more adverse events, including deep epithelial disruption, made worse if application occurred via tampon use [22]. Cervicovaginal lavages of women using LiJ for seven days showed high levels of pro-inflammatory cytokines such as IL-1, IL-6, and IL-8 and increased numbers of CD45-positive leukocytes, indicating the presence of a mucosal inflammatory response. Furthermore, a recent cross-sectional observational study of 374 FSW in Nigeria found a statistically significant association between use of LeJ/LiJ (n = 81) and the presence of cervicovaginal intraepithelial neoplasia (CIN) [23]. However, another study questioned 398 FSW about their use of LiJ/LeJ (89 LiJ/LeJ users vs 312 non-users) and related this to the prevalence of HIV and other STDs. Whilst *B. vaginosis *appeared to show an association with LiJ/LeJ usage (55.8% in users versus 44% in non-users), this did not reach statistical significance (p = 0.06). Furthermore, there were no associations between use of citrus douching and other STDs, nor the prevalence of HIV-1 infection between LiJ/LeJ users (48.8%) and non-users (48.2%) [24]. However, neither of these studies was able to control for frequency of condom use, timing of LiJ/LeJ douching and the degree to which the citrus juice was diluted [23,24].

*In vitro *studies presented here suggest that an intact cervicovaginal epithelium is relatively resilient to the toxic effects of LiJ. Reconstructed cervicovaginal epithelial cultures were able to tolerate application of 50% LiJ for 30 minutes in the absence of significant toxicity. However, viability was significantly reduced following longer exposures. Furthermore, repeated exposure of an intact epithelium to only 25% LiJ resulted in a significant loss of viability. In experiments using non-polarized cervicovaginal tissue explants, the toxic nature of LiJ was more apparent, with a 30 minute exposure to 10% LiJ causing significant toxicity, suggesting that toxicity would be higher where the cervicovaginal mucosa was damaged. This effect was also observed using penile explant tissue, suggesting that male partners would also be susceptible to the toxicity of LiJ. Cervicovaginal epithelial cell monolayers were highly susceptible to LiJ induced toxicity. Other reports have also demonstrated that LiJ is extremely toxic to both cervical tissue and T cells *in vitro *[25]. Such effects would be enhanced in women with cervical ectopy (a condition common in young women where the more fragile single layered epithelium of the endocervix displaces the stratified epithelium of the ectocervix) or in the presence of pre-existing ulcerative STDs or microtrauma induced during coitus. This is emphasized by reports that up to 19% of women found the use of LiJ as a douche painful [11].

These observations have important implications for the use of LeJ/LiJ or other acidic solutions as potential intervention strategies to reduce the risk of HIV transmission. Firstly, there is no evidence to show that vaginal douching, especially if performed before sexual intercourse, can maintain a volume of LiJ in the presence of cervicovaginal secretions to provide a 1:1 ratio (equivalent to 50% LiJ) with an infectious ejaculate (2–6 ml) for greater than 5 minutes (and up to 30 minutes). Furthermore, as non-Clade B HIV isolates have been shown to be less sensitive to the effects of pH [10], more juice or longer exposure times may be required to inactivate virus from other clades of HIV. For this approach to be successful, an acidic douche must distribute evenly throughout the vaginal lumen and effectively neutralise semen at all possible portals of virus entry. If used prior to coitus, the antiviral efficacy of this approach would depend on the time period between douching and exposure to an infectious ejaculate, potential leakage of the LiJ from the vagina in the preceding interval, dilution by cervicovaginal secretions induced by sexual arousal, and the re-equilibrium of vaginal pH with time. Measurement of vaginal pH one hour after the first 100% LiJ application in the clinical safety study mentioned above revealed only a small drop in baseline pH (mean = 0.51 units), in the absence of semen, reinforcing the notion that, applied prior to intercourse, LiJ is unlikely to be effective in inactivating HIV [22]. Furthermore, animal efficacy studies have consistently demonstrated a >1000 fold difference between *in vitro *and *in vivo *activity of compounds [4]; thus it is highly likely that even neat LiJ would be far lower than the concentration required to inactivate an infectious ejaculate *in vivo*. Therefore, the use of LeJ/LiJ to prevent HIV transmission lacks biological plausibility. Furthermore, data presented here suggest that repeated application of LeJ/LiJ is likely to result in epithelial damage. While the consequences of such damage on susceptibility to HIV transmission or other STDs are unknown, they should be cause for serious concern in high risk populations.

## Conclusion

In conclusion, our preclinical evaluation of the virucidal activity and cytotoxicity of LiJ have identified potential safety concerns for the use of LiJ as a vaginal douche in individuals at risk of HIV infection. The results also fail to demonstrate biological plausibility for the use of vaginal douching with LiJ as an intervention strategy to prevent HIV transmission.

## Methods

### Cell and virus culture

C8166 and PM-1 cells (AIDS reagent project, National Institute for Biological standards and control, Potters Bar, UK) were grown in continual culture (RPMI 10% [RPMI 1640 medium supplemented with 10% fetal bovine serum, penicillin, streptomycin and L-glutamine]) and passaged every 3–4 days. Adherent ME180 monolayers (derived from a highly invasive squamous cell carcinoma of the cervix; American Type Culture Collection (ATCC), Rockville, MD) were grown in continual culture (DMEM 10% [Dulbecco's modified Eagle's Medium supplemented with 10% fetal calf serum, penicillin, streptomycin and L-glutamine]) and passaged every 3–4 days. ME180 cells were treated with 1× trypsin/EDTA (4 ml per 75 cm^2 ^flask for approximately 5 minutes) to allow detachment of cells and passage (approximately 1 in 10) twice weekly. HIV-1 strains used were grown either in phytohaemagglutinin (PHA)-stimulated peripheral blood mononuclear cells (HIV-1_BaL_), or PM-1 cells (HIV-1_BaL _and HIV-1_RF_). Cell-free viral stocks were passed through 0.2 μm pore-size filters. Infection was monitored by viral reverse transcriptase (RT) released into culture supernatants [26].

### Lime Juice (LiJ)

Sour limes were purchased at a local grocery store. They were cut in half and manually squeezed. The resulting LiJ was filtered through a Whatman number 1 paper, and residual pulp and seeds were discarded. The LiJ of twelve limes was processed in this manner, pooled, aliquoted and stored frozen at -80°C until use. We cannot exclude the possibility that some antiviral activity may have been adsorbed by the filter paper, but consider this to be highly unlikely. The pH of the LiJ was 2.4 ± 0.0 (mean ± S.D.) which falls within the normal range for sour limes (pH 2.3–3.1). The seminal plasma used in this study immediately raised the pH of LiJ, yielding values >4.0 and >5.0, respectively, at 1:4 and 1:8 lime:semen proportions.

### Supply and culture of human samples

#### (a) Cervicovaginal tissue

Ectocervical or vaginal tissue was obtained from women undergoing planned therapeutic hysterectomy at St George's, St Helier's and Kingston Hospitals (London, UK) (written consent was obtained from all tissue donors according to the local Research Ethics Committee). Cervicovaginal tissue comprising both epithelium and stroma was cut into 3 mm explants prior to culture in 96 well, flat bottom tissue culture plates in RPMI 10%, as previously described [27-30].

#### (b) Penile glans tissue

Penile tissue was obtained from gender reassignment surgery at Charing Cross Hospital (written consent was obtained from all tissue donors according to the local Research Ethics Committee). Penile glans tissue comprising both epithelium and stroma was cut into 3 mm explants and cultured as described for cervicovaginal tissue [31].

#### (c) Seminal plasma (SP)

Semen samples were obtained from healthy normozoospermic donors participating in a research protocol approved by the Eastern Virginia Medical School Institutional Review Board (IRB). Samples were centrifuged at 500 g for 10 minutes following liquefaction, and SP was collected from the supernatant. SP from different donors was pooled, aliquoted and stored frozen at -80°C until use. We have not assessed the effects of LiJ on non-liquefied semen and cannot exclude that this might behave differently.

#### Supply and culture of reconstructed cervicovaginal epithelium

Reconstructed squamous "cervicovaginal" type epithelium cultures, derived from normal human ectocervical cells (NHEC) isolated from single donor adult human ectocervical tissue, were purchased from MatTek Corporation, USA. Cultures, supplied in 24-well tissue culture inserts, were of multilayer thickness, typically 10–16 cell layers of non-cornified tissue by histology. Cultures were shipped for use at 4°C on medium-supplemented, agarose gels in 24-well plates. Typically, shipments were sent (from the USA) on a Monday, arriving at the lab (in the UK) on Wednesday afternoon. On arrival, cultures were first allowed to equilibrate to room temperature for 1 hour, and then culture inserts carefully moved into 24-wells containing 300 μl pre-warmed culture media (RPMI 10%), ensuring no transfer of the transport agarose. Cultures were then cultured overnight at 37°C ready for use the following day.

### Determination of virucidal activity

#### (a) T cell assay: solid-phase immobilisation of HIV-1

HIV was immobilised onto the solid phase of 96-well flat-bottomed tissue culture plates coated with poly L-lysine (PLL; 50 μg/ml in PBS) for 1 hour at room temperature [6]. Following washing (1 × 200 μl PBS), wells were incubated with HIV-1_RF _(50 μl; 10^3 ^× TCID_50_) for 1 hour at 37°C after which unbound virus was removed by washing (2 × 200 μl PBS). Immobilised virus, in the absence or presence of 50% SP (this was always added to the wells first), was treated with LiJ (5–50% final concentration, diluted as necessary in 0.154 M NaCl [normal saline]) for 5, 30, 60 or 120 minutes at 37°C. LiJ/SP was removed by washing (4 × 200 μl PBS) and virus cultured with C8166 (200 μl, 4 × 10^4 ^cells/well). Viral replication was determined following 7 days in culture by measurement of reverse transcriptase activity in culture supernatants [26].

#### (b) Cervicovaginal tissue assay

HIV-1_BaL _(10^4 ^× TCID_50_) was incubated with diluted LiJ (final concentrations of 50, 25, 10, 5 or 0% LiJ, diluted in RPMI 10%) for 5, 30, 60 or 120 minutes. Cervicovaginal tissue explants were then exposed to 200 μl of the virus/LiJ incubate (pre-diluted 1/10 to reduce the potentially toxic effects of LiJ to tissue) and incubated at 37°C for 2 hours. Tissue explants were then washed (4 × 200 μl PBS) and cultured for 10 days with 50% media feeds every 2–3 days. Viral infection was determined by the release of p24 antigen released into culture supernatants (p24 antigen ELISA, Beckman Coulter, carried out according to the manufacturer's protocol). To ensure the absence of infection was not due to the toxic effects of LiJ, replicate tissues were exposed to diluted LiJ alone for 2 hours and tissue viability determined by MTT dye reduction as described below.

### Toxicity of lime juice

#### (a) Toxicity to human genital tissue

Genital tissue explants (cervicovaginal or penile) were exposed to diluted LiJ (200 μl; 5–50% final concentration, diluted in RPMI 10%) for 5, 30, 60 or 120 minutes. LiJ was then removed by washing (4 × 200 μl PBS) and viability determined using the MTT (3 [4,5-dimethylthiazol-2-yl]-2,5 dipbenyltetrazolium bromide or thiazolyl blue) dye reduction method. Briefly, explants were exposed (submerged) to 0.5 mg/ml MTT for 2 hours at 37°C when live cells can reduce the MTT dye into a methanol soluble formazan product. Explants were then blotted to remove excess liquid, weighed, transferred into 1 ml methanol and incubated overnight at room temperature in the dark. The absorbance of the methanol containing the MTT-formazan product was determined at 570 nm and the % viability per mg tissue calculated by the comparison of test samples to explants not exposed to LiJ.

#### (b) Toxicity to cervical epithelial cell monolayers

ME180 cells (0.2 × 10^5 ^cells/well) were seeded into 96-well plates and cultured overnight to approximately 60% confluency. Media was removed and cells exposed to LiJ (100 μl; 5–50% final concentration, diluted in 0.154 M NaCl) for 5, 30 or 60 minutes at 37°C. Compound was then removed by washing (4 × PBS) and media containing MTT (200 μl; 0.5 mg/ml) added to each well. Following incubation at 37°C for 2 hours, media was removed and cells solubilised in 100 μl lysis buffer (98% isopropanol/2% 2 N HCl). Well contents were mixed to evenly distribute the dye, and the absorbance determined at 570 nm.

#### (c) Toxicity to a reconstructed cervicovaginal epithelium

To assess the epithelium viability following topical application of LiJ, reconstructed cervicovaginal epithelium cultures were exposed to a topical application of LiJ (100 μl; 5–50% final concentration, diluted in 0.154 M NaCl) for 5–120 minutes. LiJ was then carefully removed by washing (2 × 200 μl PBS) and epithelium viability determined using the method of MTT dye reduction. Briefly, cultures were exposed to MTT (0.5 mg/ml in RPMI 10%; 300 μl) from the basolateral surface and incubated for 2 hours at 37°C. Cultures were then transferred into culture plates containing 1 ml methanol and the insert also filled with 1 ml methanol (such that there was 2 ml total/well, totally immersing the insert). Cultures were incubated for 2 hours (room temperature), before the dye was released by breaking the culture insert membrane and mixing the methanol to obtain a homogeneous solution. Sample absorbance was determined at 570 nm using methanol as a blank. % viability of the lime treated samples was determined by comparing to a media only treated control culture.

To determine any cumulative effect following repeat topical exposure, reconstructed cervicovaginal epithelial cultures were exposed to LiJ (5, 10 or 25% final concentration, diluted in 0.154 M NaCl) either repeatedly over 1 day, or once daily for 5 days. In each case, cultures were exposed to LiJ for 30 minutes after which LiJ was removed by washing (4 washes with PBS). Epithelia were then cultured in the absence of LiJ for either 1 hour or overnight. This was repeated for a total of five LiJ applications and epithelial viability was determined immediately following removal of the fifth application as described above.

### Statistical analyses

50% inhibitory concentration (IC_50_) and toxic dose (TD_50_) analysis was completed using non-linear regression analysis (Graphpad PRISM, GraphPad Software, Inc.). Data was also analysed by ANOVA (with Bonferroni post tests) to determine the effect of LiJ treatment on samples, and to determine the effect of the presence of SP on LiJ activity.

## Abbreviations

HIV: Human immunodeficiency virus;

AIDS: Acquired immunodeficiency syndrome;

STD: Sexually transmitted disease; 

LiJ/LeJ: Lime/lemon juice; 

SP: Seminal plasma.

## Competing interests

The author(s) declare that they have no competing interests.

## Authors' contributions

PSF participated in the design of the study, carried out cytotoxicity determinations in cellular and tissue models, the virucidal determinations using cellular assays and helped draft the manuscript. SJH carried out virucidal determination using tissue explants and the repeat exposure cytotoxicity determinations. ARB prepared the LiJ and SP, tested their pH and physical properties, and ran neutralization assays. GFD and RJS conceived the study, participated in its design and helped to draft and edit the manuscript. All authors read and approved the final manuscript.
